# 2-Amino-4-methyl­pyridinium 3-carb­oxy-4-hy­droxy­benzene­sulfonate monohydrate

**DOI:** 10.1107/S1600536810028539

**Published:** 2010-07-24

**Authors:** Madhukar Hemamalini, Hoong-Kun Fun

**Affiliations:** a X-ray Crystallography Unit, School of Physics, Universiti Sains Malaysia, 11800 USM, Penang, Malaysia

## Abstract

In the crystal structure of the title salt, C_6_H_9_N_2_^+^·C_7_H_5_O_6_S^−^·H_2_O, the water mol­ecule acts as an acceptor of bifurcated N—H⋯O hydrogen bonds from the pyridinium H atom and one H atom of the 2-amino group, forming an *R*_2_^1^(6) ring. The 3-carb­oxy-4-hy­droxy­benzene­sulfonate anions self-assemble *via* O—H⋯O hydrogen bonds, leading to supra­molecular chains along the *a* axis. These chains and *R*_2_^1^(6) motifs are linked *via* O—H⋯O, N—H⋯O and C—H⋯O hydrogen bonds, forming a layer parallel to the *ac* plane. There is also an intra­molecular O—H⋯O hydrogen bond in the 3-carb­oxy-4-hy­droxy­benzene­sulfonate anion, generating an *S*(6) ring motif.

## Related literature

For details of sulfonates, see: Onoda *et al.* (2001 ▶); Baskar Raj *et al.* (2003 ▶); Ma *et al.* (2003**a* ▶,*b* ▶,*c* ▶,*d* ▶,e* ▶). For hydrogen-bond motifs, see: Bernstein *et al.* (1995 ▶). For bond-length data, see: Allen *et al.* (1987 ▶). For the stability of the temperature controller used in the data collection, see: Cosier & Glazer (1986 ▶).
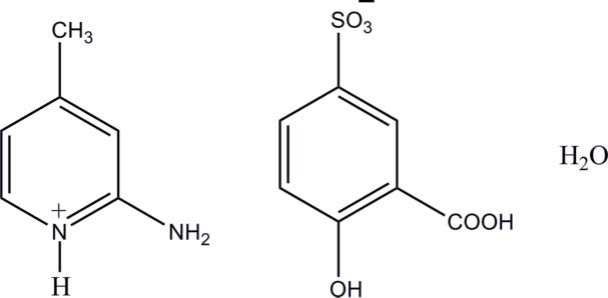


## Experimental

### 

#### Crystal data

C_6_H_9_N_2_^+^·C_7_H_5_O_6_S^−^·H_2_O
*M* *_r_* = 344.34
Monoclinic, 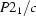

*a* = 8.3280 (9) Å
*b* = 24.122 (3) Å
*c* = 7.9355 (8) Å
β = 112.800 (3)°
*V* = 1469.6 (3) Å^3^
*Z* = 4
Mo *K*α radiation
μ = 0.26 mm^−1^
*T* = 100 K
0.22 × 0.13 × 0.04 mm

#### Data collection

Bruker APEXII DUO CCD area-detector diffractometer
Absorption correction: multi-scan (*SADABS*; Bruker, 2009 ▶) *T*_min_ = 0.945, *T*_max_ = 0.989
19925 measured reflections
5266 independent reflections
3749 reflections with *I* > 2σ(*I*)
*R*_int_ = 0.054

#### Refinement

*R*[*F* ^2^ > 2σ(*F* ^2^)] = 0.044
*wR*(*F* ^2^) = 0.120
*S* = 1.03
5266 reflections
272 parameters
H atoms treated by a mixture of independent and constrained refinement
Δρ_max_ = 0.48 e Å^−3^
Δρ_min_ = −0.46 e Å^−3^

### 

Data collection: *APEX2* (Bruker, 2009 ▶); cell refinement: *SAINT* (Bruker, 2009 ▶); data reduction: *SAINT*; program(s) used to solve structure: *SHELXTL* (Sheldrick, 2008 ▶); program(s) used to refine structure: *SHELXTL*; molecular graphics: *SHELXTL*; software used to prepare material for publication: *SHELXTL* and *PLATON* (Spek, 2009 ▶).

## Supplementary Material

Crystal structure: contains datablocks global, I. DOI: 10.1107/S1600536810028539/is2579sup1.cif

Structure factors: contains datablocks I. DOI: 10.1107/S1600536810028539/is2579Isup2.hkl

Additional supplementary materials:  crystallographic information; 3D view; checkCIF report

## Figures and Tables

**Table 1:** Hydrogen-bond geometry (Å, °)

*D*—H⋯*A*	*D*—H	H⋯*A*	*D*⋯*A*	*D*—H⋯*A*
O1*W*—H1*W*1⋯O4^i^	0.87 (2)	1.96 (2)	2.8108 (18)	168 (2)
O1*W*—H2*W*1⋯O2	0.86 (2)	2.04 (2)	2.8882 (18)	175 (2)
O1—H1*O*1⋯O2	0.89 (3)	1.84 (2)	2.6325 (18)	146 (2)
O3—H1*O*3⋯O6^ii^	0.84 (3)	1.76 (3)	2.5842 (18)	166 (2)
N1—H1*N*1⋯O1*W*^iii^	0.92 (3)	1.96 (2)	2.808 (2)	153 (2)
N2—H1*N*2⋯O1*W*^iii^	0.87 (3)	2.16 (3)	2.923 (2)	147.7 (19)
N2—H2*N*2⋯O5^i^	0.86 (3)	2.19 (3)	3.030 (2)	163 (3)
C2—H2*A*⋯O3^iv^	0.94 (2)	2.58 (2)	3.507 (2)	169.1 (17)
C4—H4*A*⋯O4^i^	0.96 (2)	2.56 (2)	3.449 (2)	155.4 (16)
C4—H4*A*⋯O5^i^	0.96 (2)	2.57 (2)	3.370 (2)	142 (2)

Symmetry codes: (i) 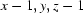; (ii) 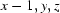; (iii) 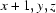; (iv) 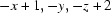.

**Fig. 1.**
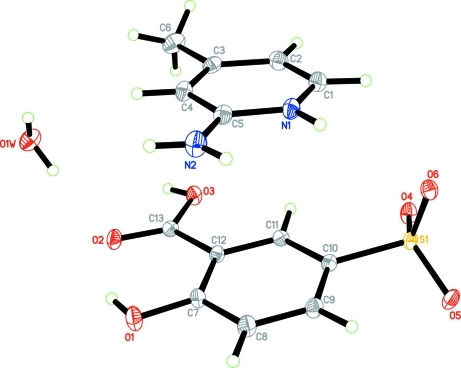
The asymmetric unit of the title compound. Displacement ellipsoids are drawn at the 50% probability level.

**Fig. 2.**
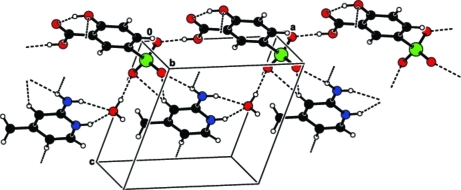
Hydrogen bonding patterns in compound (I).

## supplementary crystallographic information

### Comment

Hydrogen-bonding patterns involving sulfonate groups in biological systems and metal complexes are of current interest (Onoda *et al.*, 2001). Such interactions can be utilized for designing supramolecular architectures (Baskar Raj *et al.*, 2003). The crystal structure of transition metal (Mn, Co, Ni, Zn and Cu) complexes of the sulfosalicylate ion (3-carboxy-4-hydroxybenzenesulfonate) have been reported in the literature (Ma *et al.*, 2003**a*,*b*,*c*,*d*,e*).  Since our aim is to study some interesting hydrogen bonding interactions, the crystal structure of the title compound (I) is presented here.

The asymmetric unit of (I) contains one 2-amino-4-methylpyridinium cation, one 3-carboxy-4-hydroxybenzenesulfonate anion and a water molecule (Fig. 1). The 2-amino-4-methylpyridinium cation is planar, with a maximum deviation of 0.005 (2) Å for atom C4. The protonated N1 atom has lead to a slight increase in the C1—N1—C5 angle to 122.70 (14)°.  The bond lengths (Allen *et al.*, 1987) and angles are within normal ranges.

In the crystal packing (Fig. 2), atom O1W of the water molecule act as acceptors of bifurcated N1—H1N1···O1W and N2—H1N2···O1W hydrogen bonds with the protonated nitrogen atom and one of the 2-amino group hydrogen atom (H1N2), forming a ring with graph-set notation *R*^1^_2_(6). The 3-carboxy-4-hydroxybenzenesulfonate anions self-assemble *via* O3—H1O3···O6 hydrogen bonds, leading to a one-dimensional supramolecular chain along the *a*-axis. Furthermore, this chain and the *R*^1^_2_(6) motif are cross-linked *via* O—H···O, N—H···O and C—H···O hydrogen bonds, forming a layer parallel to the *ac* plane. There is an intramolecular O1—H1O1···O2 hydrogen bond in the 3-carboxy-4-hydroxybenzenesulfonate anion, which generates an *S*(6) ring motif.

### Experimental

A hot methanol solution (20 ml) of 2-amino-4-methylpyridine (27 mg, Aldrich) and sulfosalicylic acid (54 mg, Merck) were mixed and warmed over a heating magnetic stirrer hotplate for a few minutes. The resulting solution was allowed to cool slowly at room temperature and crystals of the title compound appeared after a few days.

### Refinement

All the H atoms were located from a difference Fourier map and refined freely [C—H = 0.94 (3)–1.013 (19) Å; N—H = 0.86 (3)–0.91 (3) Å and O—H = 0.84 (3)–0.89 (2) Å].

### Crystal data

**Table:**

C_6_H_9_N_2_^+^·C_7_H_5_O_6_S^−^·H_2_O	*F*(000) = 720
*M**_r_* = 344.34	*D*_x_ = 1.556 Mg m^−^^3^
Monoclinic, *P*2_1_/*c*	Mo *K*α radiation, λ = 0.71073 Å
Hall symbol: -P 2ybc	Cell parameters from 3564 reflections
*a* = 8.3280 (9) Å	θ = 2.9–31.3°
*b* = 24.122 (3) Å	µ = 0.26 mm^−^^1^
*c* = 7.9355 (8) Å	*T* = 100 K
β = 112.800 (3)°	Plate, colourless
*V* = 1469.6 (3) Å^3^	0.22 × 0.13 × 0.04 mm
*Z* = 4	

### Data collection

**Table:**

Bruker APEXII DUO CCD area-detector diffractometer	5266 independent reflections
Radiation source: fine-focus sealed tube	3749 reflections with *I* > 2σ(*I*)
graphite	*R*_int_ = 0.054
φ and ω scans	θ_max_ = 32.5°, θ_min_ = 1.7°
Absorption correction: multi-scan (*SADABS*; Bruker, 2009)	*h* = −12→11
*T*_min_ = 0.945, *T*_max_ = 0.989	*k* = −34→36
19925 measured reflections	*l* = −11→12

### Refinement

**Table:**

Refinement on *F*^2^	Primary atom site location: structure-invariant direct methods
Least-squares matrix: full	Secondary atom site location: difference Fourier map
*R*[*F*^2^ > 2σ(*F*^2^)] = 0.044	Hydrogen site location: inferred from neighbouring sites
*wR*(*F*^2^) = 0.120	H atoms treated by a mixture of independent and constrained refinement
*S* = 1.03	*w* = 1/[σ^2^(*F*_o_^2^) + (0.056*P*)^2^ + 0.3252*P*] where *P* = (*F*_o_^2^ + 2*F*_c_^2^)/3
5266 reflections	(Δ/σ)_max_ = 0.001
272 parameters	Δρ_max_ = 0.48 e Å^−^^3^
0 restraints	Δρ_min_ = −0.46 e Å^−^^3^

### Special details

**Table:**

Experimental. The crystal was placed in the cold stream of an Oxford Cryosystems Cobra open-flow nitrogen cryostat (Cosier & Glazer, 1986) operating at 100.0 (1) K.
Geometry. All s.u.'s (except the s.u. in the dihedral angle between two l.s. planes) are estimated using the full covariance matrix. The cell s.u.'s are taken into account individually in the estimation of s.u.'s in distances, angles and torsion angles; correlations between s.u.'s in cell parameters are only used when they are defined by crystal symmetry. An approximate (isotropic) treatment of cell s.u.'s is used for estimating s.u.'s involving l.s. planes.
Refinement. Refinement of F^2^ against ALL reflections. The weighted R-factor wR and goodness of fit S are based on F^2^, conventional R-factors R are based on F, with F set to zero for negative F^2^. The threshold expression of F^2^ > 2σ(F^2^) is used only for calculating R-factors(gt) etc. and is not relevant to the choice of reflections for refinement. R-factors based on F^2^ are statistically about twice as large as those based on F, and R- factors based on ALL data will be even larger.

### Fractional atomic coordinates and isotropic or equivalent isotropic displacement parameters (Å^2^)

**Table:**

	*x*	*y*	*z*	*U*_iso_*/*U*_eq_	
N1	0.72197 (18)	0.06878 (6)	0.66805 (18)	0.0152 (3)	
N2	0.5709 (2)	0.13358 (6)	0.4490 (2)	0.0198 (3)	
C1	0.7310 (2)	0.02604 (7)	0.7835 (2)	0.0178 (3)	
C2	0.5828 (2)	0.00495 (7)	0.7925 (2)	0.0175 (3)	
C3	0.4197 (2)	0.02782 (6)	0.6816 (2)	0.0150 (3)	
C4	0.4152 (2)	0.07114 (7)	0.5679 (2)	0.0151 (3)	
C5	0.5695 (2)	0.09223 (6)	0.5591 (2)	0.0143 (3)	
C6	0.2550 (2)	0.00524 (8)	0.6887 (2)	0.0210 (3)	
S1	1.05075 (5)	0.122233 (15)	1.12957 (5)	0.01150 (9)	
O1	0.45088 (16)	0.26192 (5)	0.70255 (16)	0.0191 (2)	
O2	0.25121 (14)	0.18998 (5)	0.77919 (15)	0.0163 (2)	
O3	0.38003 (15)	0.11993 (5)	0.96906 (15)	0.0149 (2)	
O4	0.99761 (15)	0.08555 (5)	1.24394 (14)	0.0162 (2)	
O5	1.19053 (14)	0.15990 (5)	1.23096 (15)	0.0169 (2)	
O6	1.08974 (14)	0.09095 (4)	0.99033 (14)	0.0143 (2)	
C7	0.5829 (2)	0.22831 (6)	0.8014 (2)	0.0133 (3)	
C8	0.7498 (2)	0.24303 (7)	0.8160 (2)	0.0160 (3)	
C9	0.8917 (2)	0.21104 (6)	0.9167 (2)	0.0146 (3)	
C10	0.86880 (19)	0.16349 (6)	1.00593 (19)	0.0117 (3)	
C11	0.70415 (19)	0.14816 (6)	0.99271 (19)	0.0118 (3)	
C12	0.55938 (19)	0.18022 (6)	0.88935 (19)	0.0115 (3)	
C13	0.3834 (2)	0.16422 (6)	0.87474 (19)	0.0123 (3)	
O1W	−0.05262 (17)	0.12872 (5)	0.54890 (17)	0.0207 (3)	
H1A	0.850 (3)	0.0128 (9)	0.855 (3)	0.031 (6)*	
H2A	0.592 (3)	−0.0256 (8)	0.870 (3)	0.020 (5)*	
H4A	0.309 (3)	0.0871 (8)	0.485 (3)	0.018 (5)*	
H6A	0.161 (3)	0.0148 (10)	0.581 (3)	0.044 (7)*	
H6B	0.239 (4)	0.0168 (10)	0.796 (4)	0.051 (7)*	
H6C	0.259 (4)	−0.0352 (11)	0.695 (4)	0.054 (8)*	
H9A	1.007 (3)	0.2204 (8)	0.925 (3)	0.019 (5)*	
H8A	0.766 (3)	0.2767 (9)	0.752 (3)	0.027 (5)*	
H11A	0.683 (3)	0.1137 (8)	1.054 (3)	0.017 (5)*	
H1W1	−0.027 (3)	0.1193 (9)	0.457 (3)	0.030 (6)*	
H2W1	0.034 (3)	0.1488 (10)	0.614 (3)	0.035 (6)*	
H1O1	0.353 (3)	0.2485 (10)	0.708 (3)	0.038 (6)*	
H1O3	0.280 (3)	0.1160 (10)	0.969 (3)	0.040 (7)*	
H1N1	0.822 (3)	0.0824 (9)	0.661 (3)	0.039 (7)*	
H1N2	0.671 (3)	0.1437 (9)	0.450 (3)	0.031 (6)*	
H2N2	0.471 (4)	0.1469 (11)	0.379 (3)	0.047 (7)*	

### Atomic displacement parameters (Å^2^)

**Table:**

	*U*^11^	*U*^22^	*U*^33^	*U*^12^	*U*^13^	*U*^23^
N1	0.0115 (6)	0.0191 (6)	0.0148 (6)	0.0004 (5)	0.0047 (5)	−0.0003 (5)
N2	0.0177 (8)	0.0222 (7)	0.0193 (7)	−0.0003 (6)	0.0070 (6)	0.0056 (5)
C1	0.0166 (8)	0.0182 (7)	0.0160 (7)	0.0020 (6)	0.0035 (6)	−0.0001 (6)
C2	0.0201 (8)	0.0166 (7)	0.0150 (7)	0.0018 (6)	0.0060 (6)	0.0015 (5)
C3	0.0159 (8)	0.0173 (7)	0.0120 (6)	−0.0027 (6)	0.0057 (6)	−0.0030 (5)
C4	0.0129 (7)	0.0191 (7)	0.0127 (7)	0.0005 (6)	0.0044 (6)	0.0004 (5)
C5	0.0148 (7)	0.0169 (7)	0.0115 (6)	0.0004 (6)	0.0053 (5)	−0.0006 (5)
C6	0.0204 (9)	0.0242 (9)	0.0205 (8)	−0.0073 (7)	0.0100 (7)	−0.0015 (6)
S1	0.00831 (17)	0.01437 (17)	0.01216 (16)	0.00001 (13)	0.00434 (12)	0.00150 (12)
O1	0.0124 (6)	0.0204 (6)	0.0239 (6)	0.0034 (4)	0.0062 (5)	0.0083 (4)
O2	0.0093 (5)	0.0196 (5)	0.0186 (5)	0.0009 (4)	0.0038 (4)	0.0017 (4)
O3	0.0100 (5)	0.0166 (5)	0.0204 (5)	−0.0003 (4)	0.0085 (4)	0.0033 (4)
O4	0.0124 (5)	0.0206 (6)	0.0162 (5)	0.0007 (4)	0.0062 (4)	0.0057 (4)
O5	0.0108 (5)	0.0196 (5)	0.0175 (5)	−0.0035 (4)	0.0023 (4)	−0.0011 (4)
O6	0.0118 (5)	0.0169 (5)	0.0166 (5)	0.0012 (4)	0.0079 (4)	0.0000 (4)
C7	0.0114 (7)	0.0147 (7)	0.0134 (6)	0.0018 (5)	0.0044 (5)	0.0023 (5)
C8	0.0132 (7)	0.0172 (7)	0.0188 (7)	−0.0005 (6)	0.0075 (6)	0.0044 (6)
C9	0.0110 (7)	0.0168 (7)	0.0169 (7)	−0.0012 (5)	0.0066 (6)	0.0017 (5)
C10	0.0109 (7)	0.0142 (6)	0.0105 (6)	0.0000 (5)	0.0046 (5)	−0.0012 (5)
C11	0.0119 (7)	0.0130 (6)	0.0119 (6)	−0.0002 (5)	0.0063 (5)	−0.0010 (5)
C12	0.0099 (7)	0.0137 (6)	0.0116 (6)	0.0004 (5)	0.0050 (5)	0.0001 (5)
C13	0.0119 (7)	0.0139 (6)	0.0121 (6)	−0.0002 (5)	0.0059 (5)	−0.0016 (5)
O1W	0.0168 (6)	0.0282 (7)	0.0184 (6)	−0.0051 (5)	0.0083 (5)	−0.0024 (5)

### Geometric parameters (Å, °)

**Table:**

N1—C5	1.352 (2)	S1—O6	1.4744 (10)
N1—C1	1.362 (2)	S1—C10	1.7598 (15)
N1—H1N1	0.91 (3)	O1—C7	1.3464 (18)
N2—C5	1.329 (2)	O1—H1O1	0.89 (2)
N2—H1N2	0.87 (2)	O2—C13	1.2354 (18)
N2—H2N2	0.86 (3)	O3—C13	1.3108 (17)
C1—C2	1.362 (2)	O3—H1O3	0.84 (3)
C1—H1A	0.98 (2)	C7—C8	1.396 (2)
C2—C3	1.413 (2)	C7—C12	1.4059 (19)
C2—H2A	0.946 (19)	C8—C9	1.378 (2)
C3—C4	1.372 (2)	C8—H8A	0.99 (2)
C3—C6	1.496 (2)	C9—C10	1.399 (2)
C4—C5	1.408 (2)	C9—H9A	0.96 (2)
C4—H4A	0.95 (2)	C10—C11	1.384 (2)
C6—H6A	0.94 (3)	C11—C12	1.399 (2)
C6—H6B	0.96 (3)	C11—H11A	1.013 (19)
C6—H6C	0.98 (3)	C12—C13	1.476 (2)
S1—O5	1.4498 (12)	O1W—H1W1	0.87 (2)
S1—O4	1.4539 (10)	O1W—H2W1	0.86 (3)
			
C5—N1—C1	122.70 (14)	O4—S1—O6	111.41 (6)
C5—N1—H1N1	117.5 (15)	O5—S1—C10	106.72 (7)
C1—N1—H1N1	119.8 (15)	O4—S1—C10	106.72 (7)
C5—N2—H1N2	117.3 (15)	O6—S1—C10	105.29 (6)
C5—N2—H2N2	117.0 (17)	C7—O1—H1O1	107.9 (15)
H1N2—N2—H2N2	126 (2)	C13—O3—H1O3	109.5 (17)
N1—C1—C2	120.18 (15)	O1—C7—C8	117.20 (13)
N1—C1—H1A	114.5 (13)	O1—C7—C12	123.15 (13)
C2—C1—H1A	125.3 (13)	C8—C7—C12	119.64 (14)
C1—C2—C3	119.59 (15)	C9—C8—C7	120.56 (14)
C1—C2—H2A	118.8 (12)	C9—C8—H8A	119.9 (13)
C3—C2—H2A	121.5 (12)	C7—C8—H8A	119.5 (13)
C4—C3—C2	118.76 (15)	C8—C9—C10	119.87 (14)
C4—C3—C6	120.69 (15)	C8—C9—H9A	121.1 (12)
C2—C3—C6	120.55 (14)	C10—C9—H9A	119.0 (12)
C3—C4—C5	121.00 (15)	C11—C10—C9	120.40 (14)
C3—C4—H4A	122.9 (12)	C11—C10—S1	120.31 (11)
C5—C4—H4A	115.9 (12)	C9—C10—S1	119.26 (11)
N2—C5—N1	119.28 (15)	C10—C11—C12	120.00 (13)
N2—C5—C4	122.94 (15)	C10—C11—H11A	122.3 (11)
N1—C5—C4	117.77 (14)	C12—C11—H11A	117.7 (11)
C3—C6—H6A	109.5 (15)	C11—C12—C7	119.53 (13)
C3—C6—H6B	112.1 (16)	C11—C12—C13	120.35 (13)
H6A—C6—H6B	112 (2)	C7—C12—C13	120.11 (13)
C3—C6—H6C	110.6 (17)	O2—C13—O3	123.31 (13)
H6A—C6—H6C	107 (2)	O2—C13—C12	122.56 (13)
H6B—C6—H6C	105 (2)	O3—C13—C12	114.11 (13)
O5—S1—O4	114.09 (7)	H1W1—O1W—H2W1	103 (2)
O5—S1—O6	111.96 (6)		
			
C5—N1—C1—C2	0.2 (2)	O6—S1—C10—C11	101.97 (12)
N1—C1—C2—C3	−0.2 (2)	O5—S1—C10—C9	43.01 (13)
C1—C2—C3—C4	−0.3 (2)	O4—S1—C10—C9	165.36 (11)
C1—C2—C3—C6	179.81 (15)	O6—S1—C10—C9	−76.13 (12)
C2—C3—C4—C5	0.8 (2)	C9—C10—C11—C12	0.1 (2)
C6—C3—C4—C5	−179.32 (14)	S1—C10—C11—C12	−177.97 (10)
C1—N1—C5—N2	−179.53 (14)	C10—C11—C12—C7	−0.8 (2)
C1—N1—C5—C4	0.2 (2)	C10—C11—C12—C13	−179.91 (13)
C3—C4—C5—N2	179.01 (14)	O1—C7—C12—C11	−178.68 (13)
C3—C4—C5—N1	−0.8 (2)	C8—C7—C12—C11	1.0 (2)
O1—C7—C8—C9	179.25 (14)	O1—C7—C12—C13	0.4 (2)
C12—C7—C8—C9	−0.4 (2)	C8—C7—C12—C13	−179.93 (13)
C7—C8—C9—C10	−0.3 (2)	C11—C12—C13—O2	−177.02 (13)
C8—C9—C10—C11	0.4 (2)	C7—C12—C13—O2	3.9 (2)
C8—C9—C10—S1	178.55 (12)	C11—C12—C13—O3	1.81 (19)
O5—S1—C10—C11	−138.89 (12)	C7—C12—C13—O3	−177.26 (12)
O4—S1—C10—C11	−16.54 (13)		

### Hydrogen-bond geometry (Å, °)

**Table:**

*D*—H···*A*	*D*—H	H···*A*	*D*···*A*	*D*—H···*A*
O1W—H1W1···O4^i^	0.87 (2)	1.96 (2)	2.8108 (18)	168 (2)
O1W—H2W1···O2	0.86 (2)	2.04 (2)	2.8882 (18)	175 (2)
O1—H1O1···O2	0.89 (3)	1.84 (2)	2.6325 (18)	146 (2)
O3—H1O3···O6^ii^	0.84 (3)	1.76 (3)	2.5842 (18)	166 (2)
N1—H1N1···O1W^iii^	0.92 (3)	1.96 (2)	2.808 (2)	153 (2)
N2—H1N2···O1W^iii^	0.87 (3)	2.16 (3)	2.923 (2)	147.7 (19)
N2—H2N2···O5^i^	0.86 (3)	2.19 (3)	3.030 (2)	163 (3)
C2—H2A···O3^iv^	0.94 (2)	2.58 (2)	3.507 (2)	169.1 (17)
C4—H4A···O4^i^	0.96 (2)	2.56 (2)	3.449 (2)	155.4 (16)
C4—H4A···O5^i^	0.96 (2)	2.57 (2)	3.370 (2)	142 (2)

Symmetry codes: (i) *x*−1, *y*, *z*−1; (ii) *x*−1, *y*, *z*; (iii) *x*+1, *y*, *z*; (iv) −*x*+1, −*y*, −*z*+2.
